# The Many Faces of Autophagy: Balancing Survival and Cell Death

**DOI:** 10.3390/biom14101268

**Published:** 2024-10-08

**Authors:** Barbara Canonico

**Affiliations:** Department of Biomolecular Sciences (DISB), University of Urbino, 61029 Urbino, Italy; barbara.canonico@uniurb.it

Autophagy and apoptosis are two fundamental biological mechanisms that may cooperate or be antagonistic, and both are involved in deciding the fate of cells in physiological or pathological conditions. The functional relationship between apoptosis (‘self-killing’) and autophagy (‘self-eating’) is intricate [1]. Under normal conditions, autophagy is kept at a basal level to maintain cellular homeostasis and preserve cell integrity by eliminating long-lived, overproduced, and aggregation-prone proteins or dysfunctional organelles such as damaged mitochondria [2]. The goal of this Special Issue is to delineate apoptosis and autophagy as interconnected processes, even overlapping processes, while defining the final cell fate, the result of the interplay of different cell death programs. Autophagy classically functions to maintain cell health during stressful conditions by targeting cytosolic components for degradation and recycling via lysosomal pathways. However, accumulating evidence also supports roles for autophagy-related genes (ATGs) in non-degradative processes, including cellular secretion. Furthermore, it is mandatory to focus on the interplay between the endocytic and autophagic pathways, underscoring the emerging connections between autophagy and EV secretion. [3,4].

This Special Issue entitled “The Many Faces of Autophagy: Balancing Survival and Cell Death” of *Biomolecules* includes a total of five contributions: three reviews and two original articles, also providing new information about the applications of flow cytometry and confocal laser-scanning microscopy in the fields of molecular, functional, and morphologic aspects of autophagy and apoptosis.

Cocchi et al. [5] demonstrated the chemopreventive profile of 6-(Methylsulfonyl) hexyl isothiocyanate (6-MITC), a compound present in Wasabia Japonica rizhome, finding that 6-MITC induced autophagy in Jurkat and HL-60 cells at the highest concentration tested and increased intracellular levels of ROS in a dose-dependent manner. Furthermore, the modulation of the autophagic process and the involvement of ROS levels as possible trigger mechanisms were specifically analyzed by flow cytometry.

Fontana et al. [6] demonstrated that the hyper-expression of ARF-T8D (Human ARF Tumor Suppressor Protein mutated at the conserved Threonine 8) inhibits autophagy in both HeLa and lung cancer cells H1299. This effect is due to the cells’ inability to elicit autophagosome formation upon T8D expression. The mutant ARF-T8D seems to disrupt critical signaling pathways involved in the initiation of autophagy, particularly through inhibition of ULK1 activation, a key regulator in autophagosome formation.

This impairment is further evidenced by the accumulation of autophagic markers (such as LC3-II in the cytosol) and a corresponding decrease in the degradation of long-lived proteins, confirming the suppression of autophagic flux. These findings suggest that ARF-T8D may interfere with cellular homeostasis and stress response mechanisms, which could have significant implications for the survival and proliferation of cancer cells under nutrient-deprived conditions.

Indeed, the review “Life and Death Decisions—The Many Faces of Autophagy in Cell Survival and Cell Death” by Ge Yu and Daniel J. Klionsky [7] considers the current knowledge on the physiological role of autophagy as well as its regulation and discusses the multiple functions of autophagy in cell survival and cell death. In fact, autophagy (or the autophagy machinery) facilitates cell survival in canonical and non-canonical ways. In canonical autophagy, two fundamental results are achieved: the degradation and recycling of cytoplasmic components. It is evident that cells use autophagy mainly for clearance and recycling functions. The autophagic process has the capability of participating in cell death and a role under pathologically relevant conditions. Autophagy-dependent cell death (ACD) is defined as a type of regulated cell death (RCD) that relies on the autophagic machinery or components thereof. In addition to autophagy-dependent cell death, autophagy also facilitates the processes of ferroptosis, FAS-driven extrinsic RCD, necroptosis, and autosis (a specific instance of autophagy-dependent cell death). Of note, the mitochondrion is one of the junction points for autophagy and RCD. The review focuses on the complexity of the interplay between autophagy and cell death: many of the genes involved in the autophagy and RCD pathways were originally named based on the pathways they were first identified within; however, there are many genes “moonlighting” in both pathways.

“The Role of Autophagy in Osteoarthritic Cartilage” [8] is a review paper introducing the concept of autophagy and its impact on osteoarthritis (OA), which is a public health issue with multiple mechanisms. Signaling pathways related to autophagy, such as AMPK-SIRT1-FOXO3A, and AMPK-ULK1, are mentioned, not neglecting long non-coding RNAs and circular RNAs that can promote or inhibit chondrocyte autophagy. In conclusion, the authors discuss the character of autophagy in OA and the process of the autophagy pathway, which can be modulated by some drugs, key molecules, and non-coding RNAs.

Ciesielska and Gajewska [9] depict in their review the role of autophagy in adipose tissue and summarize the current understanding of the effects of saturated and unsaturated fatty acids in autophagy modulation in adipocytes, further underscoring that, in obesity, the inhibition of autophagic function in adipose tissue may be regarded as beneficial in terms of lipid homeostasis and metabolic control, as it is related to reduced fat mass and improved insulin sensitivity.

The ambition of this issue of the journal is to take stock of what is happening in this field and help to address some of the many faces of autophagy. I am confident that this special number of *Biomolecules* will be useful to the readers.
